# Supplementary material for: Immunology of THymectomy And childhood CArdiac transplant (ITHACA): protocol for a UK-wide prospective observational cohort study to identify immunological risk factors of post-transplant lymphoproliferative disease (PTLD) in thymectomised children
Source: BMJ Open. 2023 Oct 20;13(10):e079582. doi: 10.1136/bmjopen-2023-079582 (PMC10603490; doi:10.1136/bmjopen-2023-079582)
Supplement: Supplementary data [file bmjopen-2023-079582supp001.pdf]

Supplementary Table 1: Clinical data collected for the ITHACA Study. ***Age at first median sternotomy is an agreed proxy where thymectomy isn't clearly documented in the patient's surgical notes.

**Category**	**Clinical data**
Baseline patient details	Age at transplant, Age at thymectomy*, Type of thymectomy (Total vs Partial), Type and date of first cardiac surgery (if not transplant), Cardiothoracic procedure at time of thymectomy, Sex, Ethnicity, Weight, Height, Comorbidities prior to transplantation, Concomitant medication at time of transplantation.
Transplant-related	Cardiac/Renal diagnosis, Indication for transplant, Blood group compatibility.
Infection-related	EBV and CMV (where available) serostatus of donor & recipient at transplant.
Immunosuppression	Induction therapy, maintenance immunosuppression drugs (dose and trough levels at each follow up visit), changes to maintenance immunosuppression drugs and indication for changes.
Complications	Opportunistic infections, Graft failure/rejection, PTLD, Mortality, documentation of "other" complications.
Follow up	Weight, Height, Additional medication, Relevant additional comments (also see categories IV & V)
